# miRNAs Expression Analysis in Paired Fresh/Frozen and Dissected Formalin Fixed and Paraffin Embedded Glioblastoma Using Real-Time PCR

**DOI:** 10.1371/journal.pone.0035596

**Published:** 2012-04-18

**Authors:** Dario de Biase, Michela Visani, Luca Morandi, Gianluca Marucci, Cristian Taccioli, Serenella Cerasoli, Agostino Baruzzi, Annalisa Pession, the PERNO Study group

**Affiliations:** 1 Department of Patologia Sperimentale, University of Bologna, Bologna, Italy; 2 Department of Ematologia e Scienze Oncologiche, University of Bologna, Bologna, Italy; 3 Department of Cancer Biology, Paul O'Gorman Cancer Institute, University College London, London, United Kingdom; 4 Anatomic Pathology of Bufalini Hospital, Cesena, Italy; 5 IRCCS Istituto delle Scienze Neurologiche di Bologna and Department of Biomedical & Neuromotor Sciences, University of Bologna, Bologna, Italy; NIH/NCI, United States of America

## Abstract

miRNAs are small molecules involved in gene regulation. Each tissue shows a characteristic miRNAs epression profile that could be altered during neoplastic transformation. Glioblastoma is the most aggressive brain tumour of the adult with a high rate of mortality. Recognizing a specific pattern of miRNAs for GBM could provide further boost for target therapy. The availability of fresh tissue for brain specimens is often limited and for this reason the possibility of starting from formalin fixed and paraffin embedded tissue (FFPE) could very helpful even in miRNAs expression analysis. We analysed a panel of 19 miRNAs in 30 paired samples starting both from FFPE and Fresh/Frozen material. Our data revealed that there is a good correlation in results obtained from FFPE in comparison with those obtained analysing miRNAs extracted from Fresh/Frozen specimen. In the few cases with a not good correlation value we noticed that the discrepancy could be due to dissection performed in FFPE samples. To the best of our knowledge this is the first paper demonstrating that the results obtained in miRNAs analysis using Real-Time PCR starting from FFPE specimens of glioblastoma are comparable with those obtained in Fresh/Frozen samples.

## Introduction

MicroRNAs (or miRNAs) are small (∼20–22 nt) non coding RNAs that modulate gene expression at a post-transcriptional level. They act by binding the target mRNAs repressing translation or regulating their degradation. Each miRNA, playing its role through perfect and nearly perfect complementarity with its target mRNAs, could regulate the expression of about a hundred of genes, influencing a large spectrum of physiological processes as different steps of cellular development, proliferation or apoptosis regulation [1].

Many of these pathways are altered in human neoplasia; in fact it has been demonstrated that miRNAs can act both as oncogenes or oncosuppressors, according to their target mRNAs [2]. In fact, in several neoplasia it has been observed that physiological miRNAs profile resulted modified [3]–[7].

Glioblastoma (GBM) is a highly malignant astrocytic glioma. It is the most frequent primary brain tumour and the most malignant neoplasm with astrocytic differentiation and correspond to WHO grade IV [8]. Histologically it is composed of poorly differentiated astrocytic tumour cells, with marked nuclear atypia, high mitotic activity, prominent microvascular proliferation and necrosis. Neverthless the progress in neurosurgery, chemio- and radiotherapy, molecular target identification for focused therapy (MGMT), the clinical history of the disease is usually short (less than one year in more than 50% of cases) [8], [9].

There are several evidences that different miRNAs could be up- or down-regulated in GBM. MiR-9/9* [10]–[12], miR-10a [13], miR10b [12], [14]–[16], miR17 [11], miR20a [11], miR-21 [11], [12], [14], [16], [17], miR26 [18], miR27a [18], miR182 [18], [19], miR-221 [12], [20]–[22], miR-222 [22] and miR-519d [16] were observed to be up-regulated in GBM (Table 1); on the contrary miR-7 [14], [23]–[25], miR-31 [14], miR34a [26], [27], miR-101 [14], [28], miR-137 [14], [16], miR-330 [14] were recognized as down-regulated (Table 1). The increasing evidence that miRNAs are involved in GBM development and progression could lead to recognise a specific miRNAs profile for this neoplasia.

**Table 1:** Name, chromosomal localization and expression level in GBM according to previously described data of miRNAs analysed in this study.

miRNA	Localization	Up/Downregulated in GBM	Reference
9/9*	1q22	UP	[10]–[12]
10a	17q21.32	UP	[13]
10b	2q31.1	UP	[12], [14]–[16]
17	13q31.3	UP	[11]
20a	13q31.3	UP	[11]
21	17q21.31	UP	[11], [12], [14], [16], [17]
26	3p22.2	UP	[18]
27a	19p13.13	UP	[18]
182	7q32.2	UP	[18], [19]
221	Xp11.3	UP	[12], [20]–[22]
222	Xp11.3	UP	[22]
519d	19q13.42	UP	[16]
7	9q21.3	DOWN	[14], [23]–[25]
31	9p21.3	DOWN	[14]
34a	1p36.22	DOWN	[26], [27]
101	1p31.3	DOWN	[14], [28]
137	1p21.3	DOWN	[14], [16]
330	19q13.32	DOWN	[14]

It has been demonstrated that, differently from mRNA, integrity of miRNAs is not influenced by fixation in formalin [29], probably due to their short length and to the complex Argonaute protein-miRNA [30]. The comparison of miRNAs expression starting from Fresh/Frozen or FFPE (formalin fixed and paraffin embedded) material was performed in culture cells [31] and in several tissues as prostate [32], [33], breast [34]–[36], kidney [29], [37], [38], lymphatic tissue [39], [40], tonsils [37], melanocytic nevi [41], colon carcinoma [38] and in one case of oligodendroglioma [42]. All these papers have demonstrated that there was a good correlation in miRNAs expression analysis starting both Fresh/Frozen and FFPE tissue. None of them, except for Nonn et al. [32], performed dissection in Fresh/Frozen or FFPE material. Most of miRNAs expression studies in GBM were performed on Fresh/Frozen tissue or cell lines. In central nervous system neoplasia, starting from FFPE tissue could be very useful because of archival material is readily available and follow-up is often known.

Aim of this study was to investigate the expression of 19 miRNAs in GBM starting from both Fresh/Frozen and FFPE-dissected tissues. In these last samples, the dissection allowed to enrich (>90%) the analysed material of neoplastic cells, limiting the eventual contamination due to “normal near the tumour” fraction (e.g. lymphocytes, stroma, not neoplastic glial and neuronal cells). In this way we would to investigate the feasibility of miRNAs expression analysis starting from FFPE tissues in GBM, looking for eventually differences between not dissected Fresh/Frozen samples and FFPE-dissected tissues.

## Materials and Methods

### Ethic Statement

The study was approved by Ethic Committee of Azienda Sanitaria Locale di Bologna (number of study 08075, protocol number 139/CE of 5^th^ February 2009, Bologna, Italy). All patients signed a written consent for molecular analysis and for anonymous data publication for scientific studies and all information regarding the human material used in this study was managed using anonymous numerical codes.

### Selection of Cases

Thirty cases of GBM were selected for miRNAs expression analysis from cases collected at Bellaria (institute of Anatomia Patologica, Bologna, Italy) and Bufalini (institute of Anatomia Patologica, Cesena, Italy) Hospitals, within PERNO (Progetto Emiliano-Romagnolo di Neuro-Oncologia) project. All specimens were primary GBM, and patients had not undergone neoadjuvant therapy before surgery. Patients were 14 males and 16 females, aged from 42 to 75 years (mean 63.3 ys).

The specimens were collected no longer than 45 minutes after removal and immediately a snap-frozen section was performed and the material evaluated by a pathologist in order to verify if the tissue was represented by a “high-grade glioma”.

A sample of tissue was then incubated in RNA later solution (Applied Biosystem, Austin, TX, U.S.A.) for 1 hour at room temperature and stored at −80°C after quick-frozen in liquid nitrogen. The remaining specular tissue was formalin fixed and paraffin embedded for routine histological diagnosis. All 30 samples were diagnosed as GBM according the 2007 WHO criteria [8].

Cell lines of prostate carcinoma (LNCaP, CRL-1740), breast adenocarcinoma (MCF7, HTB-22) and glioblastoma (U-87 MG, HTB14), provided by American Type Culture Collection (ATCC, Rockville, MD, USA), were used for evaluating efficiency of primers per each miRNA analysed.

### miRNAs extraction

The “Fresh/Frozen” specimens and cell lines were processed for miRNAs extraction protocol using *mir*Vana miRNA isolation kit (Applied Biosystem, Austin, TX, U.S.A.). Briefly, small RNA fraction was exctracted and enriched starting from 50 to 80 mg of tissue or 3 millions of cells according to manufacturer's protocol.

The haematoxylin and eosin (H&E) sections from FFPE specimens were reviewed by a pathologist (GM) to select the more informative block. Four 20 µm-thick sections were cut followed by one H&E control slide. The tumour area selected for the analysis was marked on the control slide to ensure, whenever possible, greater than 90% content of neoplastic cells (avoiding necrosis and lymphocytes). The four 20 µm-thick sections were manually dissected under microscopic guidance according to area selected on H&E and incubated in xylene for 3 minutes at 50°C and, after two rinses with ethanol, miRNAs were extracted using RecoverAll Total Nucleic Acid Isolation kit (Ambion, Austin, TX, U.S.A.), according to manufacturer's instructions.

Quality and quantity of smallRNAs extracted from both Fresh/Frozen and FFPE-dissected tissue were evaluated using the Agilent 2100 Bioanalyzer (Agilent Technologies, Waldbronn, Germany) and the Qubit fluorometer (Invitrogen, Carlsbad, CA, U.S.A.).

cDNA was obtained after a polyadenylation step and retrotranscription were performed using SuperScript III RT enzyme and a Universal RT Primer according to NCode miRNA first-strand cDNA synthesis and qRT-PCR Kit protocol (Invitrogen, Carlsbad, CA, U.S.A.).

### miRNAs analysis

Nineteen miRNAs (Table 1) were selected for analysis, according to their role in cancer and data previously published in literature at beginning of the study [10]–[12], [14], [16]–[18], [20], [21], [24], [25], [27]. miR103, RNU49 and U54 were used as endogenous controls.

Each forward primers used correspond to mature miRNA sequence according to miRBase database (http://microrna.sanger.ac.uk) (Table 2). Primers were modified with LNA (Locked Nucleic Acid) substitutions for increasing specificity and discriminating between miRNAs with a single base different nucleotide sequences (e.g. miR-10a and miR-10b, Table 2). Universal reverse primer was provided by NCode miRNA first-strand cDNA synthesis and qRT-PCR Kit (Invitrogen, Carlsbad, CA, U.S.A.).

**Table 2:** Name, localization and forward primer sequence of analysed miRNAs.

miRNA	Fw Primer Sequence
**hsa-miR-7**	TGGAA**G**ACTA**G**TGATTTTGTT
**hsa-miR-9**	TCTTTG**G**TTATCTAG**C**TGTATG
hsa-miR-9*	ATAAAG**C**TA**G**ATAA**C**CGAAAG
**hsa-miR-10a**	ACC**C**TGTAGA**T**CCGAATTTG
**hsa-miR-10b**	ACC**C**TGTAGA**A**CCGAATTTG
**hsa-miR-17**	CAAAGTGCTTA**C**A**G**TGCAG
**hsa-miR-20a**	TAAAGTGCTTA**T**A**G**TGCAG
**hsa-miR-21**	TAG**C**TTATCA**G**ACTGATGTTG
**hsa-miR-26a**	CAAGTAAT**C**CAGGATA**G**GC
**hsa-miR-27a**	TTCA**C**AGTGGCTAAGTTC**C**
**hsa-miR-31**	AGGCAA**G**ATGCTGG**C**ATA
**hsa-miR-34a**	TGG**C**AGTGT**C**TTAGCTG
**hsa-miR-101**	TACAGTA**C**TGT**G**ATAACTGAA
**hsa-miR-137**	TTATTG**C**TTAAGAATA**C**GCGT
**hsa-miR-182**	TTTGGCA**A**TGGTAGAA**C**TCAC
**hsa-miR-221**	GCTA**C**ATTGTCTG**C**TGGGTT
**hsa-miR-222**	GCTA**C**ATCTGG**C**TACTGG
**hsa-miR-330**	TCT**C**TGGGCCTGTGT**C**TTA
**hsa-miR-519d**	AAGTGC**C**TCC**C**TT**T**AGAGT

LNA bases are underlined. Fw: forward. Hsa: Homo sapiens (human).

Efficiency of each primer was tested by Real-Time PCR using serial dilutions (1∶1, 1∶25, 1∶50, 1∶100) of a pool of RNA extracted by following cell lines: U-87 MG, MCF7 and LNCaP. A run of Real-Time PCR using as template a pool of female DNA (Promega, Madison, WI, U.S.A.) was performed to confirm that miRNAs primers were not able to amplify DNA.

miRNAs expression was evaluated using a AB7000 machine (Applied Biosystem, Foster City, CA, USA) and FastStart Taq Reagents Kit (Roche, Mannheim, Germany), with the following program: 2 minutes at 50°C, 4 minutes at 95°C and 37 cycles with annealing at 60°C for 30 seconds. GelStar stain (Lonza Bioscience, Rockland, ME, USA) was used as Real-Time detector. No template control for each miRNA was included in the reaction plate. All the reactions were performed in duplicate and amplicons run on a 3% agarose gel.

### Statistical analysis

Expression values and fold-change were obtained by relative quantification and 2^−ΔΔCt^ method [43], using DataAssist 2.0 Tool (Applied Biosystem, Foster City, CA, USA). Statistical analysis of miRNAs expression was performed using GraphPad Prism 5.0 tool. Paired samples comparison and correlation analysis between miRNAs expression in Fresh/Frozen and FFPE-dissected samples were performed using Wilcoxon paired test and Spearman correlation respectively. Level of significance was p<0.05 for all the statistical analysis.

## Results

Distribution for Fresh/Frozen (FF) and FFPE samples was found not normal, as demonstrated by the Shapiro Test (p<0.001). For this reason, we only used non-parametric statistical tests.

**Figure 1:**
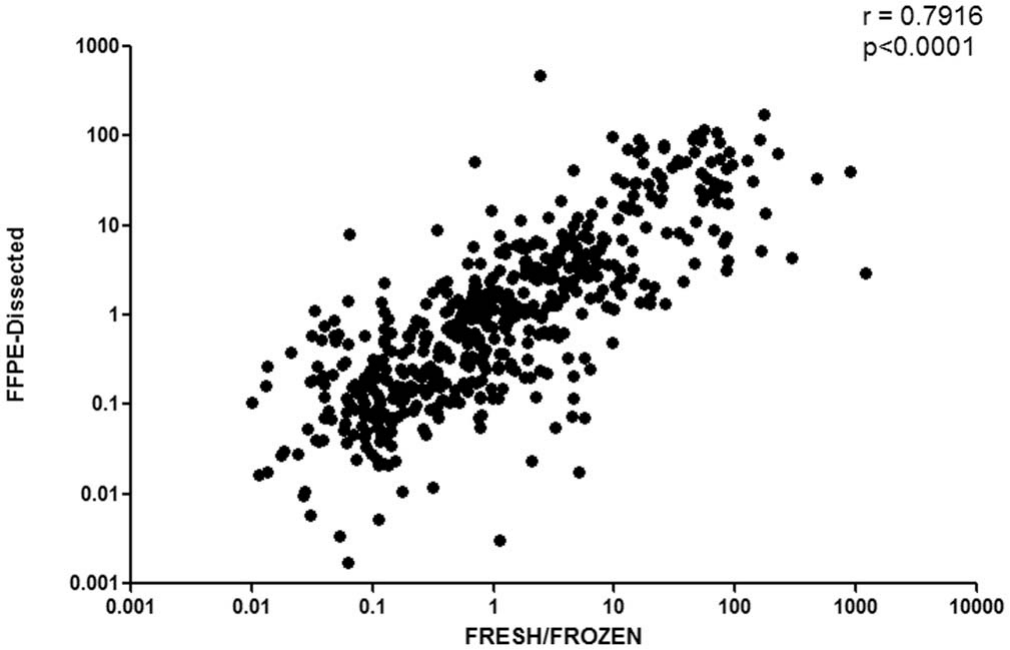
Scatter plot showing Spearman correlation between Fresh/Frozen and FFPE-dissected groups.

A good Spearman correlation value (r = 0.7916, p<0.0001) between the expression level of each miRNAs comparing results obtained in fresh-frozen and in FFPE-dissected samples was observed (Figure 1) whereas Wilcoxon paired test showed not significant differences between the two groups (p = 0.1845).

**Figure 2:**
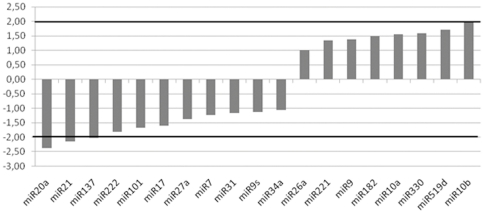
Median fold-change calculated per each miRNA between 30 paired Fresh/Frozen and FFPE samples. The y-axis represents the fold-change value.

To test if the miRNAs profile obtained in Fresh/Frozen and FFPE-dissected (FD) samples were comparable, we calculated the median fold-change for each 30 FF samples versus 30 FD specimens. Although miR-137, miR-20a and miR-21 were slightly downregulated (FD/FF ratio <−2.0), the vast majority of miRNAs were not statistically significantly different (Figure 2)

**Figure 3:**
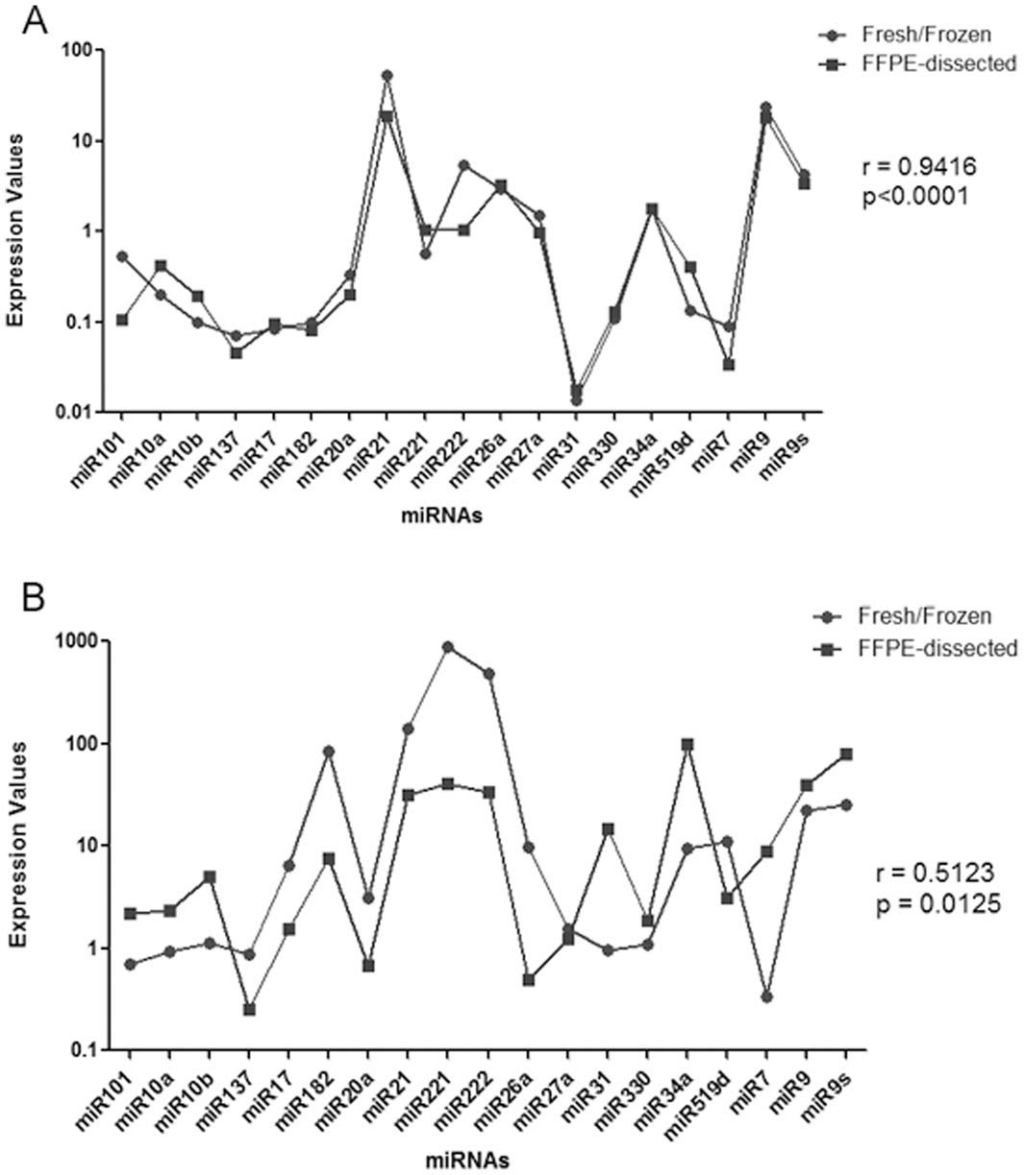
Comparison between miRNAs expression profile in Fresh/Frozen and in FFPE specimens. a) Example of one specimen with a good correlation of miRNAs expression profile obtained in Fresh/Frozen (pointed line) and in FFPE specimen (squared line); b) Example of one specimen with a correlation less than r<0.65 of miRNAs expression profile obtained in Fresh/Frozen (pointed line) and in FFPE specimen (squared line).

Comparison of individual miRNAs expression between Fresh/Frozen and FFPE-dissected sample, in single paired specimen, showed a good Spearman correlation value (r>0.65) in 25 out of 30 samples (Figure 3a) while the remaining five cases showed a correlation ratio <0.65 (ranged from 0.5123 to 0.6386, Figure 3b).

To investigate if discrepancy observed in the 5 cases with r<0.65 could be caused by enrichment in neoplastic cells due to dissection, we analysed the miRNAs profiles of these 5 samples starting from undissected FFPE material. We performed the analysis only in the 4 cases in which the H&E revealed the presence of not-neoplastic tissue adjacent the area dissected for miRNAs analysis (Table 3). In 3 out of 4 cases analysed the Spearman correlation value increased up the cut off of 0.65 (Table 3).

**Table 3:** Spearman correlation values between miRNA profiles obtained in Fresh/Frozen, FFPE-dissected and FFPE-not dissected samples.

Case	R (Fresh/Frozen vs FFPE-dissected)	R (Fresh/Frozen vs FFPE-not dissected)	Composition of not-dissected FFPE sample	
			Neoplastic cells (%)	Not-neoplastic cells (%)
1	0.51	NP	98	2
2	0.60	0.70	50	50
3	0.50	0.38	75	25
4	0.62	0.81	70	30
5	0.63	0.89	40	60

NP: Not Performed.

## Discussion

The use of formalin-fixed paraffin embedded samples for nucleic acid analysis in molecular study gives more disposal of specimen for research. For this reason, miRNAs analysis starting from FFPE samples could be of great usefulness for miRNAs expression study. Due to their short length (19–25 nt), the mature miRNAs seem not to be influenced by nucleic acid degradation caused by formalin fixation [29], as happened on the contrary for long RNA or DNA. Several papers reported the feasibility of miRNAs expression from FFPE specimens in different tissues as kidney, prostate and breast [32], [33], [35]–[38].

GBM is the most aggressive adult brain tumour and, nevertheless the progresses in molecular therapy, its prognosis remains very poor [8]. Identifying a miRNAs profile for GBM could be very useful for better clarify prognosis and researching new targeted drugs. For this reason, and for “opening” the anatomic pathology archives even to analysis of miRNAs expression in GBM, it is crucial determining if FFPE specimens are suitable for this type of analysis.

Our study demonstrated, in a cohort of 30 paired GBM, that miRNAs analysis using real-time technique could be performed starting from FFPE samples as well as from Fresh/Frozen specimens. The data demonstrated that there is a good correlation (r = 0.7916) between the profiles obtained starting from FFPE-dissected samples and from fresh samples.

The real cellular composition of Fresh/Frozen sample is not well known, in fact, even if a 4 µm-thick snap-frozen section was used for evaluating fresh sample, the miRNAs extraction was performed starting from 50–80 mg of not morphologically checked tissue (containing, for example, lymphocytes or non-neoplastic cells). This situation could lead to discrepant results in miRNAs analysis that we observed in 5 out of 30 cases here analysed. In FFPE-dissected samples, the selection of area used for performing the analysis lead to enrich the sample in neoplastic cells, avoiding “contamination” due to non-tumoural components. In 3 out of 4 cases, with a not good (r<0.65) Spearman correlation value, the analysis of miRNAs expression performed without dissection resulted in a better correlation with corresponding Fresh/Frozen samples. In only one case the correlation coefficient value remained below 0.65, even when obtained without dissecting the sample. To our knowledge, this sample did not show peculiar histological features (i.e. predominant lymphocytic infiltrate or necrotic zone).

To the best of our knowledge, this is the first study comparing the miRNAs expression analysis in GBM in FFPE-dissected samples and Fresh/Frozen specimens.

Our data demonstrated that in a cohort of 30 GBM, as happened in other tissues, data of miRNAs expression analysis are comparable starting from FFPE sample as well as from Fresh/Frozen specimens. This approach have several advantages: it is possible to check the real composition of the analysed sample, and it could be possible to dispose of archival material for miRNAs expression analysis (even considering the difficult to retrieve fresh brain tissue). The fact that dissection could influence the expression results leads to put a lot of attention in comparing miRNAs analysis performed with or without dissection.
